# Drugs Modulating Renin-Angiotensin System in COVID-19 Treatment

**DOI:** 10.3390/biomedicines10020502

**Published:** 2022-02-21

**Authors:** Jose L. Labandeira-Garcia, Carmen M. Labandeira, Rita Valenzuela, Maria A. Pedrosa, Aloia Quijano, Ana I. Rodriguez-Perez

**Affiliations:** 1 Research Center for Molecular Medicine and Chronic Diseases (CIMUS), IDIS, University of Santiago de Compostela, 15782 Santiago de Compostela, Spain; carmen.maria.labandeira.guerra@sergas.es (C.M.L.); rita.valenzuela@usc.es (R.V.); mary.pedrosa@usc.es (M.A.P.); aloia.quijano.ocampo@usc.es (A.Q.); 2 Networking Research Center on Neurodegenerative Diseases (CIBERNED), 28031 Madrid, Spain; 3 Neurology Service, Hospital Alvaro Cunqueiro, University Hospital Complex, 36213 Vigo, Spain

**Keywords:** angiotensin, ACE2, ACEI, ARB, AT2 agonists, COVID-19 therapy, ibuprofen, mas receptor agonists, RAS inhibitors, SARS-CoV-2

## Abstract

A massive worldwide vaccination campaign constitutes the main tool against the COVID-19 pandemic. However, drug treatments are also necessary. Antivirals are the most frequently considered treatments. However, strategies targeting mechanisms involved in disease aggravation may also be effective. A major role of the tissue renin-angiotensin system (RAS) in the pathophysiology and severity of COVID-19 has been suggested. The main link between RAS and COVID-19 is angiotensin-converting enzyme 2 (ACE2), a central RAS component and the primary binding site for SARS-CoV-2 that facilitates the virus entry into host cells. An initial suggestion that the susceptibility to infection and disease severity may be enhanced by angiotensin type-1 receptor blockers (ARBs) and ACE inhibitors (ACEIs) because they increase ACE2 levels, led to the consideration of discontinuing treatments in thousands of patients. More recent experimental and clinical data indicate that ACEIs and, particularly, ARBs can be beneficial for COVID-19 outcome, both by reducing inflammatory responses and by triggering mechanisms (such as ADAM17 inhibition) counteracting viral entry. Strategies directly activating RAS anti-inflammatory components such as soluble ACE2, Angiotensin 1-7 analogues, and Mas or AT2 receptor agonists may also be beneficial. However, while ACEIs and ARBs are cheap and widely used, the second type of strategies are currently under study.

## 1. Introduction

The 2019 coronavirus pandemic (COVID-19) was caused by SARS-CoV-2, which is a member of a family of RNA viruses that infect humans, mainly producing respiratory tract infections and respiratory distress syndrome. However, although respiratory symptoms are predominant, multiorgan dysfunction related to a more generalized inflammatory response can occur in severe cases. In most cases, COVID-19 infection is asymptomatic or almost asymptomatic. However, in a small percentage of cases, the disease can be severe or fatal, particularly in elders and patients with preexisting diseases such as diabetes, hypertension, and heart disease [1]. WHO Clinical Progression Scale considers five categories for the patient’s state: uninfected (score 0), ambulatory mild disease (score 1–3), hospitalized moderate disease (score 4–5), hospitalized severe disease (score 6–9), and death (score 10). Overall, COVID-19 has caused millions of deaths and devastating health, social, and economic effects in all countries.

A massive worldwide vaccination campaign appeared to be the best solution. However, this is being complicated by difficulties in the vaccination of part of the population and in carrying out vaccination in developing countries, by the spread of the new variants, and by problems with vaccine efficacy [2,3]. Drug treatments are clearly necessary, particularly in most vulnerable patients. However, most current treatments are still experimental. Antivirals are the most frequently considered treatments, although usually with more disappointment than success. However, strategies targeting mechanisms involved in the aggravation of COVID-19 may also be useful. 

A considerable number of studies have suggested the major role of the tissue renin-angiotensin system (RAS) in the pathophysiology and severity of COVID-19 [4,5,6,7,8]. The link between RAS and COVID-19 is mainly related to the role of angiotensin-converting enzyme 2 (ACE2), a central component of the RAS, as the primary binding site for SARS-CoV-2 that facilitates the virus entry into host cells [9,10]. ACE2 plays a key role in balancing the tissue RAS against the anti-inflammatory response, which is supported by several previous studies that have shown the protective role of ACE2 and its product Angiotensin 1-7 (Ang 1-7) in experimental lung lesions [11,12]. However, ACE2 is also the SARS-CoV-2 entry receptor [13,14], which initially suggests that the increase in ACE2 expression may lead to an increase in cell infection. This raised the initial dilemma of whether we should increase ACE2 levels in tissues to inhibit the inflammatory response, or promote the reduction of tissue ACE2 levels to decrease viral entry and replication. This has been termed as the double-edged sword of ACE2 [15,16,17]. The complexity of the question is further increased because several studies have shown that viral binding reduces the ACE2 levels at the cell surface [18,19], which may shift the RAS balance toward inflammation, fibrosis, and progression of disease severity. Altogether, this suggests that ACE2 targeting by SARS-CoV-2 is not just the entry route of the viral infection but may trigger a major mechanism of COVID-19 severity by promoting tissue RAS dysregulation, which induces a hyperinflammatory state in several organs, leading to lung injury, hematological alterations, and immunological dysregulation.

A collateral consequence of this apparent double-edged sword role for ACE2 was an important medical controversy regarding the use of angiotensin-converting enzyme inhibitors (ACEIs) and angiotensin type-1 receptor blockers (ARBs). These drugs may increase the tissue levels of ACE2 according to some studies [20,21] and are the treatment of millions of patients with chronic diseases, especially those particularly vulnerable to COVID-19, such as elders, diabetics, obese, and cardiovascular and hypertensive patients [22,23]. 

An initial hypothesis, widely disseminated through the media, suggesting that the susceptibility to infection and disease severity may be enhanced by these treatments led to serious concerns and the consideration of discontinuing these drugs at the beginning of the pandemic [24,25,26]. However, observational studies performed in the following months could not find any increase in the risk or severity of COVID-19 related to these RAS inhibitors [27,28], giving support to recommendations of Health Care Organizations and Scientific Societies that advised not to discontinue treatments to avoid risks due to the worsening of the underlying diseases. More recent studies have provided data on the potential benefit of angiotensin receptor modulators in COVID-19 [29,30,31], and in a recent experimental study, we have shown molecular mechanisms that support these potential beneficial effects of ARBs and ACEIs in COVID-19 treatment [32]. In the present review article, we will analyze representative studies on RAS organization and, particularly, RAS-related treatments in COVID-19. In addition to the results reported by recent clinical observations and clinical trials, we will analyze experimental data from our laboratory and others that mechanistically support the results of clinical studies.

## 2. The Renin-Angiotensin System

RAS components were observed for the first time in kidney extracts more than a century ago [33], and it is known that RAS is one of the oldest hormone systems in vertebrate phylogeny [34,35]. The RAS was originally considered a circulating hormonal system related to the regulation of blood pressure and sodium and water homeostasis. Then, local or paracrine RAS were observed in many tissues, which play a major role in tissue physiology and pathophysiology. Both the circulating RAS and paracrine RAS may act together in most tissues. However, the hormonal RAS appears less relevant than the local paracrine RAS for the tissue function [36]. 

**Figure 1:**
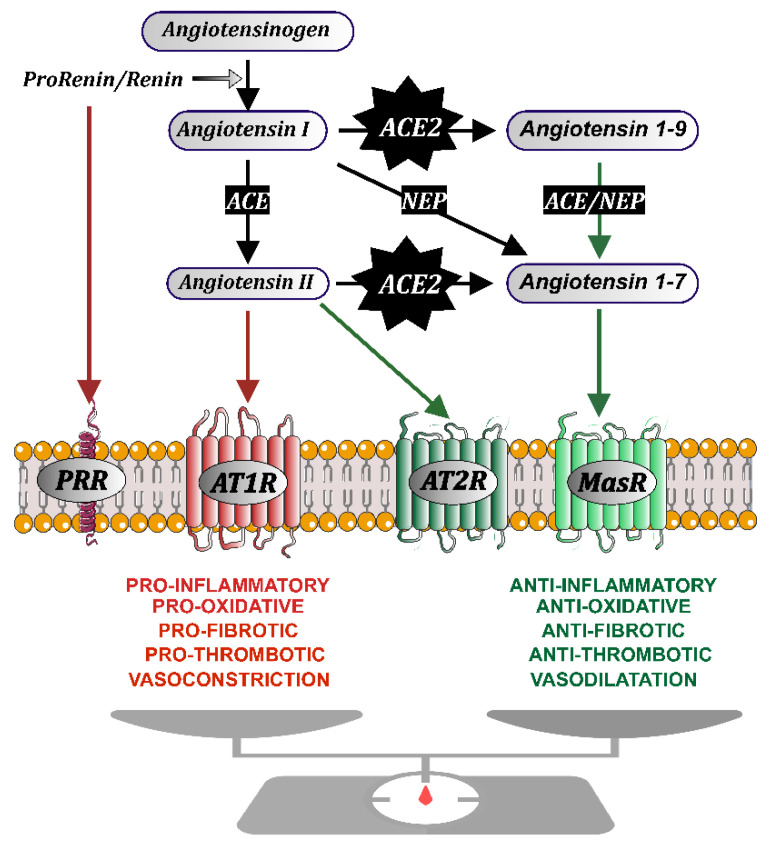
The renin-angiotensin system (RAS) consists of two axes that counteract each other: a pro-inflammatory axis (red arrows) mainly constituted by Angiotensin II acting on AT1 receptors (AT1R), and an anti-inflammatory axis (green arrows) constituted by Angiotensin II acting on AT2 receptors, and particularly Angiotensin 1-7 acting on Mas receptors. Angiotensin II is produced by the action the enzyme prorenin/renin on the precursor protein angiotensinogen, producing Angiotensin I, which is transformed by the angiotensin-converting enzyme (ACE) into Angiotensin II. Renin and its precursor prorenin (PR) can also activate specific PR receptors. Angiotensin-converting enzyme 2 (ACE2) plays a central role in the RAS balance, as ACE2 (with the aid of peptidases such as Neprilysin, NE) converts compounds of the pro-inflammatory arm (Angiotensin I and, particularly, Angiotensin II) into compounds of the anti-inflammatory arm (i.e., Angiotensin 1-9 and, particularly Angiotensin 1-7).

The classical main RAS effector peptide is angiotensin II (Ang II), which is generated by the action of two enzymes, renin and angiotensin-converting enzyme (ACE), sequentially acting on the precursor glycoprotein angiotensinogen (Figure 1). Ang II acts on two major G-protein coupled receptors: Ang II type 1 and 2 (AT1 and AT2) receptors. AT2 receptor effects are usually opposed to effects exerted by AT1 receptors [37]. Overactivation of tissue RAS, via AT1 receptors, leads to oxidative stress and inflammatory processes, which play a major role in the tissue inflammatory response and aging-related degenerative changes [38,39], including the release of pro-inflammatory cytokines, such as IL-1, IL-6, and TNF-α, and macrophage activation [40]. Ang II/AT1 activation of the cell membrane NADPH-oxidase complex 2 (Nox2) induces the intracellular generation of superoxide and superoxide-derived reactive oxygen species (ROS), which play a major role in the oxidative and inflammatory effects of Ang II/AT1 activity [41,42,43]. 

More recently, a number of additional peptides, enzymes, and receptors (among which ACE2, Ang 1-7, and Mas receptors can be highlighted) were observed to modulate the RAS function by counter-regulating the above-mentioned classical prooxidative pro-inflammatory RAS. Thus, the RAS is basically organized into two axes that counteract each other and must be tightly balanced in physiological conditions [7,44]: a pro-inflammatory and prooxidative arm mainly formed by Ang II and AT1 receptors, and an anti-inflammatory antioxidative arm formed by Ang II/AT2 receptors and Ang 1-7/Mas receptors. Interestingly, ACE2 plays a central role in RAS balance, because ACE2 converts compounds of the pro-inflammatory arm (such as Ang I and particularly Ang II) into compounds of the anti-inflammatory arm (such as Ang 1-9 and particularly Ang 1-7) (Figure 1).

In addition to the classical circulating/hormonal RAS and the paracrine or tissular RAS, the complexity of the system has been increased by the observation of an intracellular/intracrine RAS in several cell types including fibroblasts, vascular smooth muscle cells, cardiac cells, kidney cells, and neurons [45,46,47]. Interestingly, we have recently observed that an ACE2/Mas-related axis is highly expressed in the mitochondria [48]. 

## 3. ACEIs, ARBs and COVID-19: Clinical Studies

ACEIs and ARBs are widely used for treatment of several cardiovascular diseases, such as hypertension and ischemic heart disease. ACEIs decrease formation of Ang II from its precursor Ang I, while ARBs act by blocking the action of Ang II on its prooxidative/pro-inflammatory receptor AT1. Therefore, both types of drugs downregulate the activity of the RAS pro-inflammatory axis. However, ACEIs can produce additional effects, such as a decrease in the effects of Ang II on the anti-inflammatory receptor AT2, and particularly, ACEIs can also induce an increase in bradykinin, which has been related with coughing and inflammation of the respiratory tract. Bradykinin is a vasoactive peptide involved in blood pressure regulation and inflammation by increasing vascular permeability and vasodilatation. Bradykinin’s pro-inflammatory effects may counteract some aspects of the ACEI-induced downregulation of the Ang II/AT1 pro-inflammatory axis, making ACEIs less effective against the inflammatory response than ARBs [49,50]. Consistent with the wide distribution of paracrine RAS, effects of ACEIs and ARBs have been observed in different organs and particularly in the lung, where they improved fibrosis and severe lung injuries both in mouse models of lung injury and different viral pneumonias in patients [51,52,53], suggesting their possible beneficial effects in COVID-19 [10,54]. However, the above-mentioned possible effects of these drugs (i.e., inhibition of the pro-inflammatory RAS and enhancement of the anti-inflammatory RAS) also may imply an increase in the expression of the virus entry receptor (ACE2), leading to a particular complex situation in the case of COVID-19.

As a consequence of the above-mentioned initial alarm on the possible increase in risk of infection and severity of COVID-19 in the numerous patients using these drugs, a considerable number of observational studies, meta-analyses, and systematic reviews evaluating possible association between RAS inhibitors and COVID-19 were published or preprinted over the last year, including open-access documents for updating on this issue [55,56]. Although these studies had limitations [57], most of them showed that the use of ACEIs/ARBs is not associated with more risk of infection or increase in severity of infected patients [27,28,58,59,60]. Furthermore, several randomized controlled trials such as BRACE-CORONA and REPLACE COVID also showed the safety of the use of these drugs in COVID-19 patients [61,62]. 

Moreover, a series of studies reported less COVID-19 severity and better clinical outcomes in patients treated with ACEIs or ARBs [63,64,65], although several of these studies showed limitations like those observed in studies reporting no association. Interestingly, in an open multicenter randomized clinical trial using telmisartan, treated patients had lower median time-to-discharge and reduced death by day 30 than untreated patients [30]. The final analysis of the international HOPE COVID-19 (Health Outcome Predictive Evaluation for COVID-19), that included 6503 patients, was particularly interesting as a number of confounders observed in previous studies were subjected to multivariate adjustment [31]. In COVID-19 hospitalized patients, this study revealed that ARBs/ACEIs use until admission was associated with worse prognosis than controls after a non-adjusted analysis, probably related to their older age, clinical profiles and comorbidities; however, when adjusted for the potential bias, the historic use of ARBs/ACEIs at admission showed similar outcomes in both cohorts. However, considering only the in-hospital use of these drugs and after adjusting for all relevant bias, the in-hospital use of ARBs/ACEIs was associated with an important prognostic benefit, including survival [31].

## 4. ACEIs and ARBs: Effects on Tissue ACE2 Levels

**Figure 2:**
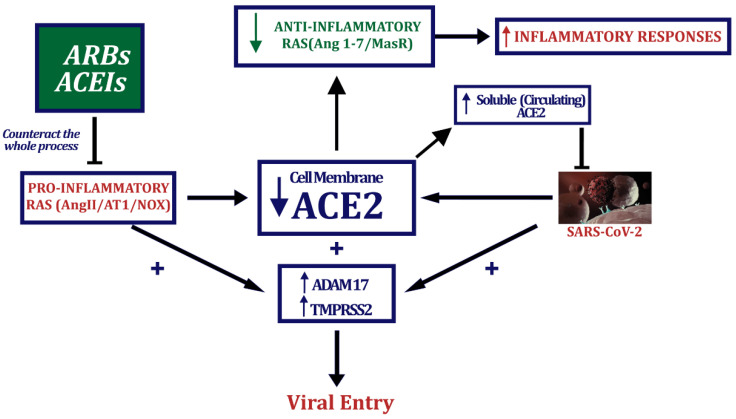
Both SARS-CoV-2 binding to cell membrane ACE2 and activation of the RAS pro-inflammatory arm decrease levels of membrane ACE2 and increase ADAM17 and TMPRSS2 activities. A decrease in transmembrane ACE2 activity leads to a decrease in anti-inflammatory RAS activity. Treatment with ARBs or ACEIs reduces the activity of the pro-inflammatory RAS axis, upregulating ACE2 transmembrane levels and the anti-inflammatory axis activity. Inhibition of the RAS pro-inflammatory axis also inhibits ADAM17 and TMPRSS2 activities, which are necessary for membrane ACE2 shedding and viral entry. Membrane ACE2 shedding further reduces membrane ACE2 and releases circulating soluble ACE2 that may bind/neutralize circulating viruses.

The effect of ARBs and ACEIs on lung levels of ACE2 has been controversial, which became particularly relevant for patients vulnerable to severe COVID-19, such as aged patients and patients with obesity, hypertension, or diabetes taking these drugs. However, most of the controversy was based on review or opinion articles that at the same time highlighted that experimental data on this question were scarce and controversial [66,67]. In healthy animals, there were a couple of studies showing increase in ACE2 expression in the heart and kidney [20,21], but there were no data of the effects of these drugs in the lung of animals or humans. A few more data were reported on the effects of these drugs on animal models of diseases, such as cardiovascular or renal diseases [68,69,70,71,72], usually showing an increase in ACE2 expression or normalization of decreased ACE2 expression. Furthermore, in addition to their effect on ACE2 expression, ACEI, or ARB effects on the functioning of other RAS components may affect the balance of the RAS pro-inflammatory/anti-inflammatory arms and play a major role in COVID-19 severity (Figure 2).

To clarify this issue, particularly in the lung, we recently studied the effects of the ARB candesartan and the ACEI captopril on lung levels of ACE2 and other major RAS components in adult healthy rats, and in aged rats and rats with metabolic syndrome (obesity, increased blood pressure, hyperglycemia) as animal models of individuals particularly vulnerable to severe COVID-19 [32]. In cultures of human alveolar type-II pneumocyte cells, we studied the effects of both types of drugs on changes induced by viral spike protein on ACE2 levels [32]. We observed that both drugs up-regulated ACE2 expression in the lung of healthy rats, and also induced the up-regulation of other major elements of the RAS anti-inflammatory arm such as AT2 and Mas receptors. This suggests that the use of ACEIs or ARBs promotes the anti-inflammatory RAS axis in the lung leading to anti-inflammatory, antithrombotic, and antifibrotic responses that may contribute to a better outcome of COVID-19 (Figure 2). In aged rats and rats with metabolic syndrome, we observed a decrease in lung levels of ACE2 and a decrease in the expression of components of the anti-inflammatory axis such as AT2 and Mas receptors, together with a significant increase in AT1 receptor expression, which indicated a clear imbalance towards the pro-inflammatory RAS in the lung. In these models of vulnerable patients, treatment with captopril or candesartan induced a significant increase in the expression of lung ACE2, AT2 and Mas receptors, and a decrease in the expression of AT1 receptors. Pro-inflammatory receptor (AT1) expression was decreased to levels of healthy controls, and the expression of anti-inflammatory receptors such as AT2 and, particularly, Mas was upregulated to levels higher than those observed in healthy untreated controls, revealing a shift in the RAS balance towards the anti-inflammatory axis [32]. Our observations in cultures of human alveolar type-II pneumocytes supported the results in animal models since we observed that treatment with candesartan or captopril restored levels of transmembrane ACE2 decreased by treatment of cells with the viral spike protein [32]. 

ACE2 upregulation by ARB/ACEI treatment appears related to several mechanisms. First, there is an increase in mRNA ACE2 expression as indicated above. Second, Ang II, via AT1 receptor activation, promotes a reduction of transmembrane ACE2 from the cell surface (shedding) by activation of metalloprotease ADAM17, leading to the increase in the soluble form of ACE2 and internalization of ACE2-derived polypeptides [73,74], as detailed below (Figure 2). However, additional mechanisms may also be involved. In a recent study, we observed that ACE2 and other RAS receptors form functional complexes at the cell surface and that ligand binding to AT1 receptor induced the downregulation of transmembrane ACE2 expression, while ligand binding to AT2 receptors induced upregulation of ACE2 [75].

## 5. ACEIs and ARBs: Effects on SARS-CoV-2 Entry

Experimental data indicate that these drugs increase levels of transmembrane ACE2, which appears beneficial by shifting the tissue RAS towards the anti-inflammatory axis. Therefore, the main point is what happens in the presence of SARS-CoV-2, and whether these treatments, which increase the viral receptor ACE2, enhance virus entry and infection. SARS-CoV-2 enters the cells mainly using the spike protein, which includes two subunits, S1 and S2. S1 binds the surface cell receptor (ACE2) and S2 mediates cell membrane fusion with the virus [14,76,77]. The SARS-CoV-2–ACE2 complex is mainly internalized by endocytosis resulting in viral entry and reduction of cell surface ACE2 [9,10,78]. ADAM17 is a metalloprotease and disintegrin located in the cell membrane that cleaves the ectodomain of ACE2 into the extracellular space [79,80,81] and its inhibition decreased virus replication revealing that shedding is necessary for viral entry [79]. Activation of ADAM17 by the viral spike protein depends on the ACE2 cytoplasmatic domain, which is involved in viral entry [79,82]. The ACE2 cytoplasmic domain is also necessary for ACE2 shedding induced by the viral spike protein [79,82]. The soluble/circulating ACE2 form can constitute a complex with the virus, leading to reduction in viral load at the cell surface [83]. The transmembrane protease serine 2 (TMPRSS2) also plays a relevant role in cell infection by participating in membrane fusion and internalization of the spike-ACE2 complex, essential for host cell infection [9] (Figure 2).

In cultures of human alveolar type-II pneumocytes treated with the spike protein and the ARB candesartan or the ACEI captopril, we observed that up-regulation of ACE2 by these drugs may be counteracted by parallel mechanisms leading to reduction of SARS-CoV-2 spike protein entry, particularly by inhibition of ADAM17 activity [32]. Consistent with this, cultures treated with spike protein and candesartan or captopril showed a decrease in the entry of spike protein inside cells as observed by laser confocal microscopy. Cultures of human alveolar type-II pneumocytes treated with spike protein showed a decrease in cell levels of transmembrane (i.e., full-length) ACE2, together with an increase in soluble ACE2 in the culture medium and increase in cell levels of a short ACE2 polypeptide, which may correspond to an internalized glycosylated ACE2 polypeptide. These effects of spike on ACE2 were inhibited by pretreatment with candesartan or captopril, suggesting a reduction in spike/ACE2 internalization and supporting the decrease observed with confocal microscopy. This is also consistent with a candesartan/captopril-induced decrease in ADAM17 activity observed in our cultures, and with previous studies showing that Ang II, via AT1/Nox-derived ROS, increased ADAM17 activity in cardiomyocytes and neurons [84,85], (Figure 2). 

Our studies on pro-inflammatory cytokine release also support a beneficial effect of candesartan and captopril [32]. As observed in other studies [86,87], treatment of human alveolar type-II pneumocytes with spike protein induced an increase in the release of pro-inflammatory cytokines such as TNF-α, IL-6 and the chemokine CCL2 to culture medium. This is consistent with that observed in sera of SARS-CoV-infected patients [88,89] and the supernatant of a SARS-CoV-infected culture systems [86,87,89,90]. The release of pro-inflammatory cytokines induced by treatment with spike protein was decreased when cultures were also treated with candesartan or captopril. Spike induced up-regulation of the pro-inflammatory AT1/NOX2 axis (inhibited by ARBs) can increase cytokine release [91,92] mediated by NOX2-derived ROS [84,93]. Furthermore, ADAM17 (also inhibited by ARBs) promotes the shedding of ACE2 [81,94,95], but also the shedding of several cytokines [96]. It is well-known that ADAM17, also known as TACE (TNF-α-converting enzyme), was initially related to TNF-α release from cells [97], promotes IL-6/soluble IL-6 receptor release [91], and may induce CCL2 release via indirect mechanisms [98]. 

## 6. Angiotensin Receptor Autoantibodies in COVID-19—Further Support to the Use of RAS Inhibitors (ARBs)

Several inflammatory processes have been associated with autoantibodies against RAS components. AT1 receptor autoantibodies (AT1-AA) act as AT1 receptor agonists enhancing the pro-inflammatory RAS activity in several inflammatory processes [99,100,101]. ACE2 autoantibodies (ACE2-AA) inhibit ACE2 function [102], decreasing the anti-inflammatory axis activity, also shifting the balance towards the RAS pro-inflammatory axis. The mechanisms of generation of these autoantibodies require further clarification. However, the increase in AT1 autoantibodies has been associated with the increase in levels of pro-inflammatory cytokines such as TNF-α and IL-6 [103,104], and particularly TNFSF14 (tumor necrosis factor ligand superfamily member 14, LIGHT) [105].

In a recent study, we showed that AT1-AA and ACE2-AA are associated with an increase in the severity of COVID-19 outcome, suggesting that they could be an index of probable evolution towards severity [106], which was also observed in several recent studies [107,108]. It is known that the increase in levels of pro-inflammatory cytokines plays a main role in COVID-19 severity, and we also observed that levels of AT1 autoantibodies were highly correlated with the levels of several serum cytokines, particularly LIGHT, while levels of ACE2-AA correlated with levels of AT1-AA. From these data we suggested that in COVID-19 there is an increase in levels in pro-inflammatory cytokines, including LIGHT, which induce an increase in AT1-AA (AT1 receptor agonists), promoting the pro-inflammatory RAS activity. As mentioned above, SARS-CoV-2 binding to transmembrane ACE2 induces a decrease in cell surface ACE2 and increases the levels of circulating-soluble ACE2. This is enhanced by AT1 receptor activation (i.e., by AT1-AA; see above). The decrease in transmembrane ACE2 reduces the RAS anti-inflammatory activity. Furthermore, increased levels of circulating ACE2-SARS-CoV-2 complexes may induce generation of ACE2-AA [109], which further decreases cell surface ACE2 and the anti-inflammatory RAS activity. The association of RAS autoantibodies with COVID-19 severity further support the protective role AT1 receptor blockers in COVID-19, which may disrupt this vicious circle.

## 7. Modulation of Other RAS Components for COVID-19 Treatment

**Figure 3:**
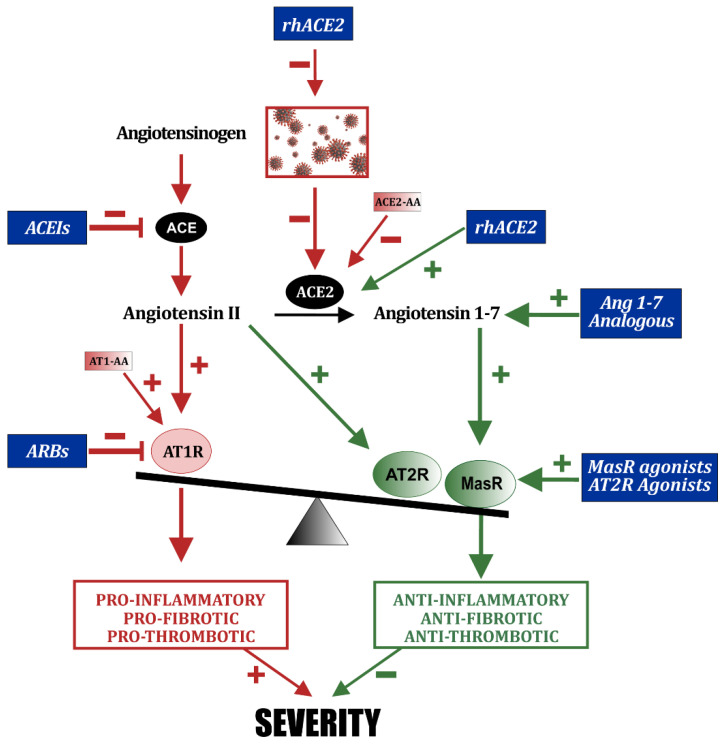
Current therapeutical strategies with drugs that inhibit components of the RAS pro-inflammatory axis or enhance components of the RAS anti-inflammatory axis.

The use of soluble ACE2 to block circulating coronavirus by binding the viral spike protein to the administered ACE2 has been suggested in several studies [110,111] (Figure 3). In vitro studies have shown that treatment with a soluble recombinant human form of ACE2 (rhACE2) led to an important reduction in the burden of SARS-CoV-2, suggesting that this strategy may neutralize SARS-CoV-2 infection [112,113,114]. It has been also suggested that administration of rhACE2 may minimize damage to organs such as lungs, kidneys, and heart by reducing Ang II concentrations and increasing conversion to Ang 1-7. The use of ACE2-like enzymes, such as B38-CAP, with structural similarity with ACE2 and that catalyze the conversion of Ang II to Ang 1-7 has been also proposed [115,116].

However, while a trap effect for circulating SARS-CoV2 leading to a decrease viral burden appears more consistent, the effects at tissue level performed by the transmembrane ACE2 appear more complicated to replicate with rhACE2, and the effects of the tissue RAS appear more important than those of the circulating RAS components for the inflammatory response in those organs. In addition, the possibility that the excess of circulating ACE2/SARS-CoV-2 complexes enhances the generation of ACE2 autoantibodies [109] should be clarified in future studies. In any case, a considerable number of clinical trials using administration of different forms of soluble ACE2 have been initiated [117] and their results will clarify the possibilities of this strategy.

As described above, ARBs and ACEIs inhibit the RAS pro-inflammatory axis and indirectly promote the RAS anti-inflammatory axis. The possibility of direct promotion of the RAS anti-inflammatory axis has also been suggested (Figure 3). Administration of ACE2 was proposed (see above), not only as a trap for circulating SARS-CoV2, but considering a possible effect on RAS function, as administration of soluble ACE2 was reported to improve lung injuries in experimental models [118]. A second possibility is the use of the ACE2 product angiotensin 1-7 (Ang 1-7) or its analogues. The use of Ang 1-7 as cardiovascular therapeutic drug has been considered some time ago. However, its use is limited by its short half-life and rapid turnover, and Ang 1-7 analogues or agonists of its receptor Mas may have more potential as therapeutic agents [119,120]. In COVID-19, several clinical trials with Ang-1-7 or Ang 1-7 analogues (such as NCT04332666, 04401423, 04375124) have been designed, as well as trials with Mas receptor agonists such as the COVA study for the agonist BIO101 [121].

As described above, a second major receptor of the anti-inflammatory or protective axis is the AT2 receptor. Therefore, the possibility of the beneficial effects of AT2 activation with AT2 agonists has also been considered. In particular, a trial with the agonist Compound 21 (C21; Vicore Pharma, Gothenburg, Sweden) has been performed in COVID-19 hospitalized patients not requiring intensive care. Patients were treated with C21 for 7 days on top of standard of care (Angiotensin II Type Two Receptor Agonist in COVID-19 Trial (ATTRACT) study; NCT04452435) [122]. Treatment with C21 was well tolerated and safe. No difference in rate of decline of their biomarker of severity (C-reactive protein) was observed relative to placebo. However, a marked reduction of requirement for oxygen at day 14 was observed in a post hoc analysis in patients treated with C21, which suggested to extend the evaluation in a Phase 3 study (NCT04880642; estimated study completion date is March 2022).

## 8. Non-Steroidal Anti-Inflammatory Drugs, ACE2 and RAS. An Early Controversy

Together with the use of ARBs and ACEIs, the use of non-steroidal anti-inflammatory drugs (NSAIDs), particularly ibuprofen, was highly controversial at the beginning of the COVID-19 pandemic [123,124]. As in the case of the above-mentioned RAS inhibitors, the origin of the controversy was related to some experimental data [125] suggesting that ibuprofen may induce an increase in the ACE2 expression in cells, which may increase the risk of viral infection and severity [16,26]. Data from clinical trials were also controversial, reporting no significant association [126] or detrimental [127] or beneficial [128] effects. The major anti-inflammatory properties of NSAIDs, such as ibuprofen, are related to COX-2 inhibition [124,129]. However, interactions between COX-2 and RAS have also been observed, particularly in kidney studies [130,131,132].

To clarify this point, we performed an experimental study with ibuprofen [133], parallel to that performed for ARBs and ACEIs [32]. Consistent with that found in the heart of streptozotocin-induced diabetic rats [125], the major source of the above-mentioned controversy, we observed that treatment with oral ibuprofen increased the expression of tissue ACE2 in the lung of adult rats. In addition, ibuprofen downregulated major components of the pro-inflammatory axis (particularly AT1 expression and Ang II levels) and upregulated RAS anti-inflammatory components (levels of Ang 1-7, and expression of AT2 and Mas receptors) in the lung, shifting the balance towards the anti-inflammatory arm. In rats with metabolic syndrome (obesity, increased blood pressure, hyperglycemia) used as a model of most vulnerable population, these effects were particularly evident [133].

Human type-II pneumocytes cultured in the presence of ibuprofen also upregulated levels of ACE2 expression. As observed with ARBs, administration of viral spike protein to pneumocyte cultures decreased levels of transmembrane ACE2 and increased the levels of soluble ACE2 and major pro-inflammatory cytokines in the culture medium. Spike administration also induced upregulation of ADAM17 and transmembrane protease serine 2 (TMPRSS2) activities in pneumocytes. As in the case of ARBs, the above-mentioned spike-induced changes, and the rate of spike protein internalization by pneumocytes were inhibited by pretreatment with ibuprofen (Figure 2).

Our observations are consistent with previous studies that proposed ADAM17 inhibitors as antiviral drugs after observing that they inhibited the internalization of pseudotyped virus expressing the SARS-spike protein and the internalization of infectious strains of SARS-CoV [82]. The inhibitory effects of ibuprofen on ADAM17 activity may be related to its inhibitory effect on the RAS pro-inflammatory axis Ang II/AT1/NADPH-oxidase, which is supported by previous studies in cardiomyocytes and neurons showing that Ang II, through AT1/NADPH-oxidase-derived ROS, induces ADAM17 activation [84,85]. An ibuprofen-induced downregulation of TMPRSS2 was previously observed in other cell types [134,135].

Our results suggest that ibuprofen induces an increase ACE2 levels and the RAS anti-inflammatory activity in lung/pneumocytes, which has been observed to be beneficial in several previous studies of experimental lung injuries [11,12]. Furthermore, the ibuprofen-induced inhibition of ADAM17 and TMPRSS2 activities may counteract effects of the increase in ACE2 on viral entry. Altogether, this suggests that ibuprofen not only can be safety used in COVID-19 but exerts a beneficial effect on the COVID-19 outcome via modulating the tissue RAS towards the anti-inflammatory axis.

## 9. Conclusions

The current data indicate that drugs that reduce RAS pro-inflammatory activity (ACEIs and, particularly, ARBs) can be beneficial for the COVID-19 outcome, both by reducing organ (particularly lung) inflammation and by triggering mechanisms (such as ADAM17 inhibition) that counteract a possible increase in viral entry related to the increase in the expression of cell ACE2. Strategies directly enhancing the activity of RAS anti-inflammatory components such ACE2, Ang 1-7, Mas, or AT2 receptors may also be beneficial. However, while ACEIs and particularly ARBs are cheap and widely used, the second type of strategies are currently under study. RAS modulators may be useful acting together with other therapeutical strategies, particularly antiviral drugs. As in the case of other drugs, the potential beneficial effects of ARBs and ACEIs can be different in different patients depending on other external factors, including combinations with other treatments, patient comorbidities, and even the sex or age of the patient. In fact, beneficial effects were more clearly observed in studies that considered possible confounding factors and relevant bias in their analysis [31,136]. Obviously, the classical limitations of these drugs, such as the risk of potential birth defects in women that may get pregnant, must also be considered. As commented on above, recent data also suggest that patients showing high levels of AT1 autoantibodies may be the most benefited candidates [106,137]. Interestingly, recent studies have suggested the use of machine learning to identify patients who may get the greatest benefits from treatment with these drugs, or patients in which the benefit may be poor or even detrimental, and algorithms to help with the evaluation of efficacy and risks of ARBs and ACEIs in COVID-19 patients have been suggested [138]. 

## Data Availability

Not applicable.
